# Features for Evaluating Source Localization Effectiveness in Sound Maps from Acoustic Cameras

**DOI:** 10.3390/s24144696

**Published:** 2024-07-19

**Authors:** Luca Fredianelli, Gregorio Pedrini, Matteo Bolognese, Marco Bernardini, Francesco Fidecaro, Gaetano Licitra

**Affiliations:** 1 National Research Council (CNR), a Moruzzi 1, 56124 Pisa, Italy; g.licitra@arpat.toscana.it; 2 Department of Earth Sciences, University of Pisa, Via Santa Maria 53, 56127 Pisa, Italy; g.pedrini1@studenti.unipi.it; 3 Environmental Protection Agency of Tuscany Region (ARPAT), Via Vittorio Veneto, 27, 56127 Pisa, Italy; 4 Ipool S.r.l., Via Enrico Fermi, 75, 51100 Pistoia, Italy; marcobernardini.s@gmail.com; 5 Physics Department, University of Pisa, Largo Bruno Pontecorvo 3, 56127 Pisa, Italy

**Keywords:** acoustic camera, beamforming algorithms, sound signals, microphone array, source localization, sound maps, environmental noise, noise measurements

## Abstract

Acoustic cameras (ACs) have become very popular in the last decade as an increasing number of applications in environmental acoustics are observed, which are mainly used to display the points of greatest noise emission of one or more sound sources. The results obtained are not yet certifiable because the beamforming algorithms or hardware behave differently under different measurement conditions, but at present, not enough studies have been dedicated to clarify the issues. The present study aims to provide a methodology to extract analytical features from sound maps obtained with ACs, which are generally only visual information. Based on the inputs obtained through a specific measurement campaign carried out with an AC and a known sound source in free field conditions, the present work elaborated a methodology for gathering the coordinates of the maximum emission point on screen, its distance from the real position of the source and the uncertainty associated with this position. The results obtained with the proposed method can be compared, thus acting as a basis for future comparison studies among calculations made with different beamforming algorithms or data gathered with different ACs in all real case scenarios. The method can be applicable to any other sector interested in gathering data from intensity maps not related to sound.

## 1. Introduction

The rapid development of computationally efficient and technological improvements has led to the current status of acoustic cameras (ACs) as the state-of-the-art microphone array evolution. These devices comprise numerous microphones that work in unison to capture sound signals simultaneously along with a video camera and a computational unit that analyzes the arrival angle of the waves. This final operation is carried out through the use of algorithms based on beamforming techniques. New instruments can now rapidly process large quantities of data, enabling real-time applications of beamforming to the acquired signal. The combination of a video camera and a microphone array allows the beamforming to visualize the origin of a sound in a video acquisition or static frame [1,2,3,4].

In recent years, ACs have been used for investigating the sound emissions of different sources: trains [5,6], cars [6,7], aircrafts [8] and helicopters [3,9,10,11], Unmanned Aerial Vehicles (UAVs) [12,13], wind turbines [14,15], ports and ships [16,17]. Other applications are in the automotive sector [18,19] or inside yachts [20] to identify sound leakages and then improve the acoustic comfort.

The increase in applications and the consequent commercialization of ACs has led to the development of different models and brands. However, all ACs create a sound map with the acquired signals according to the incident angle and overlap it with an image simultaneously taken with the video camera. The detection of a source emerging over the background implies a main lobe in the sound map due to the direct sound wave from the source. In the presence of more or less reflective surfaces along the propagation, sound waves other than the direct one are generated due to reflections and diffraction. The detection of those waves can lead to the generation of secondary lobes on the sound maps, corresponding to fake sources (also known as ghost sources). These artifacts increase the complexity of the estimation of the direction of arrival of the signals. After the first Delay and Sum (DAS) [21,22], different researchers or manufacturers have then investigated and elaborated increasingly sophisticated algorithms in order to distinguish real and fake sources. Commercial development has then led several manufacturers or research groups to work independently and produce proprietary and closed algorithms [23]. Technicians who use ACs for field measurements at present must face technical difficulties such choosing which algorithm to apply independently in different scenarios. This is a crucial step to ensure the highest reliability and optimize AC applications, as applying different algorithms to the same AC acquisition can result in varying source locations [16]. To date, there have been few comparisons between algorithms in the literature with the majority of studies conducted under test conditions. This leaves room for further research.

A method allowing the verification of the effectiveness of source localization would therefore be necessary. In the literature, few works investigated a performance evaluation procedure for ACs and different beamforming algorithms  [24] or evaluate through Monte Carlo simulations [25,26], while others focused on performance evaluation on specific experimental setups [27,28]. However, all the studies were based on the availability of raw data from the AC, which are only few times available due to the manufacturers’ choice.

In fact, to the authors’ knowledge, most ACs have different proprietary software for the analysis of acquired signals. Most of the time, the outputs are not the 2D matrix of sound pressure data but rather only the sound maps as images. The absence of values represents a difficulty for those who wish to carry out comparisons and analysis of an analytical kind.

The present study seeks a solution to this limitation by providing a methodology for extracting features from sound maps, thus passing from a simple visualization of images to numerical values that can be analyzed or used for comparisons. The objective is reached starting from measurements carried out on a test site with an omnidirectional white source, where the origin of the sound signals is then known. The features extraction algorithm, applied to any AC’s sound map, returns the difference between the location of the positioned measurement point on the image plane and the true position of the source as well as the associated uncertainty. Other visual parameters are also developed in order to better support the evaluation of beamforming algorithms not originating symmetrical maps.

The authors expect the work to have great utility because, in addition to defining specific parameters for evaluating efficiency, a method is proposed for extracting analytical information from sound maps obtained with any AC, especially for those that do not provide numerical results. The proposed features extraction algorithm would then be applicable independently from the hardware product and, in most cases, would spare computational time and facilitate sharing/transfer of data. Moreover, it would allow correlating and comparing data from different origins, thus acting as a basis for the subsequent evaluation of the effectiveness of different algorithms or ACs in various conditions.

The rest of the paper is organized as follows: in Section 2, Materials and Methods, the experimental setup is described; in Section 3, the developed feature extraction algorithm is described in detail; in Section 4, the effects of the tunable parameters on the results of the procedure are discussed. Conclusions are finally reported in Section 5.

## 2. Materials and Methods

The present paragraph describes the experimental set-up, the instrumentation specifications and a summary of the proposed features extraction algorithm with a particular focus on its inputs. Measurements were carried out with the acoustic camera available at the Physics Department of the University of Pisa. The useful AC specs are outlined below:

- Diameter of the array: 170 cm in Fibonacci spiral;
- Number of MEMS microphones: 112;
- Resolution: 24-bit;
- Sampling rate: 48 kHz;
- Frequencies range of acquisition: from 10 Hz to 24 kHz;
- Camera aperture angle: 55.2°.

According to the manufacturer’s specifications, beamforming should be reliable above 150 Hz. To be on the safe side, no calculations were made below 250 Hz. The measurements were performed in a field with a flat, grass-covered ground to reproduce a real case scenario without reflection from surfaces except from the ground. Throughout the duration of the recordings, the periods in which external sources were perceptible were avoided in order to reduce artifacts. A dodecahedron loudspeaker was used to diffuse white noise in the 20 Hz–20 kHz frequency range and fixed intensity. The AC remained in a fixed position throughout the experiment while the source was moved. Recordings were taken with the source moved at different distances and positions, including four distances and six positions for a total of twenty-four combinations. The distances were 15, 30, 45, and 60 m, respectively; left, center and right measurements were taken for each distance with the source first on the ground and then 1.2 m above it. These arrangements generated various angles of incidence for the sound rays going from the source to the AC. By measuring at those angles of incidence, the performance of the AC in identification and representing the source can be investigated and evaluated.

These measurements had the sole purpose of providing AC images to be elaborated with the developed analysis method. The signals acquired with AC were then processed using the native software provided by the manufacturer.

**Figure 1:**
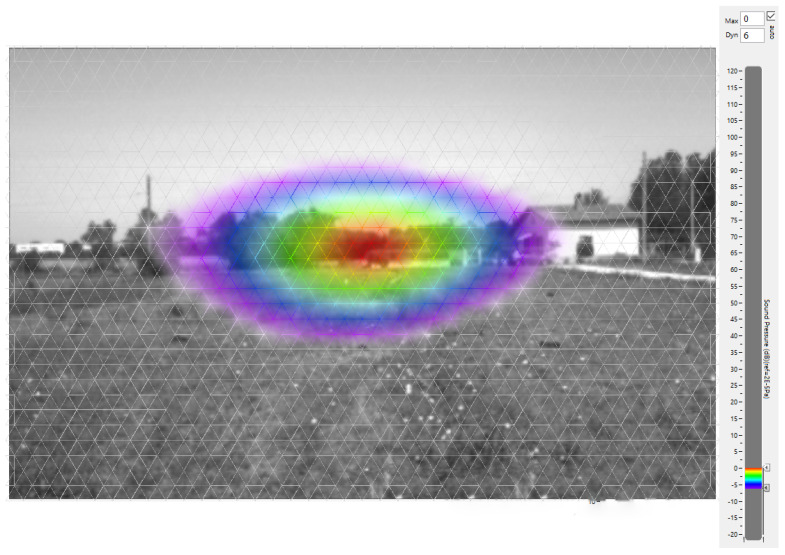
Example of output image from an acoustic camera software, which was used as input to the procedure. By convention, the software assigned 0 dB to the maximum level (red); thus, negative values are assigned to the others.

The software’s output are black-and-white images of the scene with the sound map superimposed. The values are drawn following a chromatic scale depending on the dynamic range selected by the operator. For the purpose of the present work, the images were processed with each image taking an average of six seconds over the twenty-second acquisition, with a dynamic range of 6 dB; i.e, the color scale started from the highest measured level down to minus 6 dB from the maximum measured level ($L_{max}$). Images as shown in Figure 1 are used as input images for the features extraction algorithm.

The features extraction algorithm is based on the following steps:

1. Correlation between level scale (dB) and colors in hue saturation and value (HSV).
2. Conversion to HSV and initial filtering.
3. Artifacts removal.
4. Data extraction.
5. Data definition and representation.

The following outputs of this process list the parameters defined to characterize the sound map and that would be used to evaluate beamforming algorithms and AC performances:

- *r*: distance modulus between the expected and the measured centers of the source, measured in pixels (px).
- $\sigma_{r}$: dispersion of the sound map, measured in pixels (px).
- $A_{-1dB}$: surface of sound map from $L_{max}$ to $L_{max}$ – 1 dB.
- $OVL_{-1dB}$: percentage of overlapping between the real source and the $A_{-1dB}$ mask.
- $SC_{-1dB}$: surface comparison between the real source dimension and $A_{-1dB}$ in percentage.

## 3. Features Extraction Algorithm

**Figure 2:**
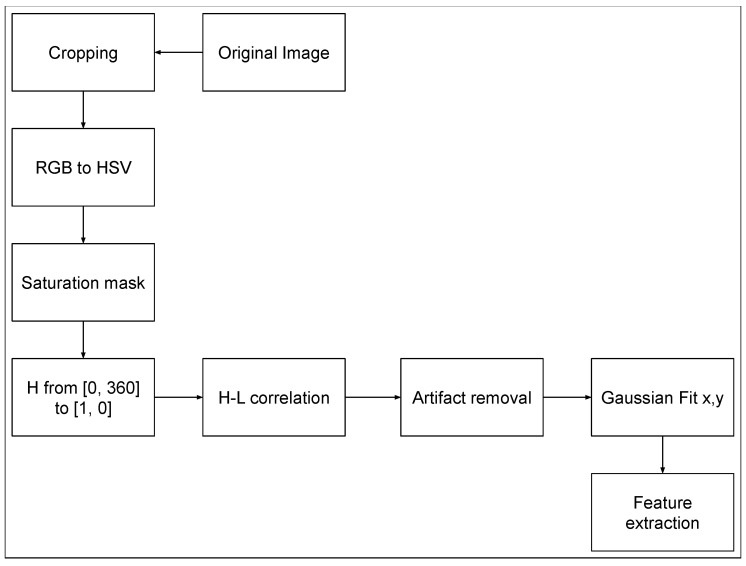
Flow chart of the feature extraction algorithm.

The present section describes the features extraction algorithm, whose inputs are the image with a superimposed sound map processed by the native software of AC. The output can be used to evaluate the real distance between the source localized on screen and the real position of the source together with its uncertainty. A flow chart of the algorithm is depicted in Figure 2.

### 3.1. dB–HSV Correlation

**Figure 3:**
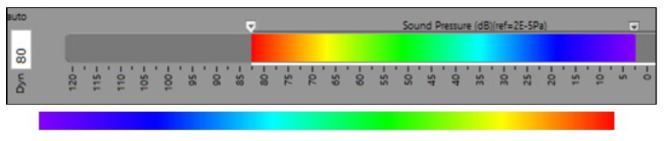
Example of dB–hue scale (**top**) and detail of the color range (**bottom**).

The procedure starts by using a dB–hue correlation scale. In the input images, the color “red” indicates the highest level measured. As the level goes down, the colors also move down on the color scale according to the dynamic range set by the user. An example is reported in Figure 3, where the dynamic range is set to 80 dB, which implies that the color scale ranges from $L_{max}$ to $L_{max}$-80 dB. The color scale used by the software may not be common to all the available AC software in the market. By the identification of the parameters connected to the sound level (*L*), the proposed approach can be applied to the vast majority of the used color pattern. In fact, the presented methodology is applicable to any color scale based on a bijective relation between the measured sound level and one of the variables of the color space (HSV, RGB, etc). For example, in the case of a grayscale, there will be a bijective relationship between value and sound pressure.

Figure 3, where the dynamic range is set to 80 dB, implies that the color scale ranges from $L_{max}$ to $L_{max}$-80 dB.

**Figure 4:**
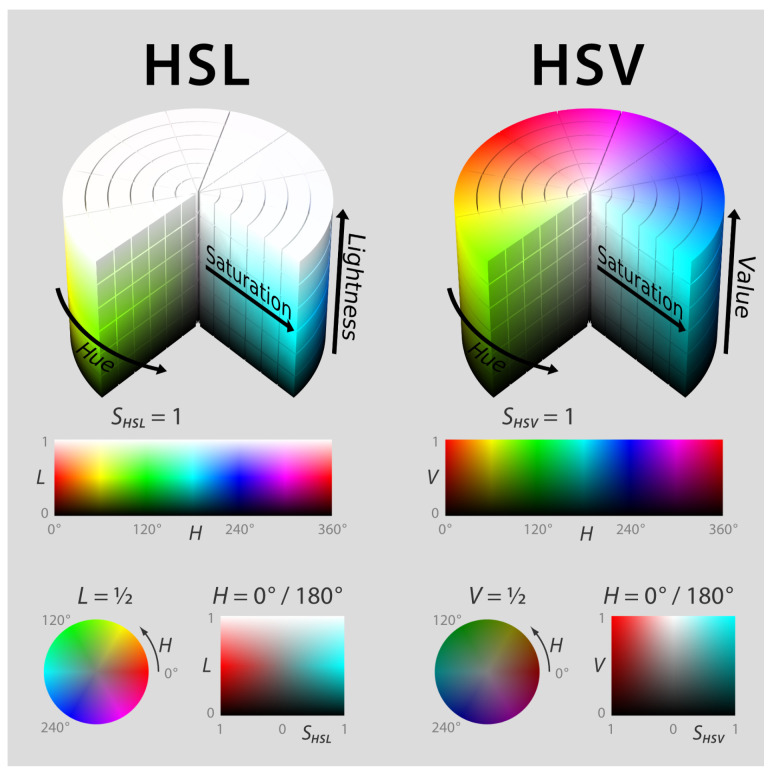
HSL and HSV color space [29].

The analyzed color scale is connected to the hue (*H*) parameter that is used in the HSL (hue, saturation, lightness) and HSV (hue, saturation, value) color representations. As reported in Figure 4, HSV represents colors in a cylindrical system of coordinates where saturation (*S*) represents the radius, volume (*V*) represents the z coordinate and *H* represents the angle associated with the color. H varies from 0° for red, through green at 120° and blue at 240°, and then back to red at 360°.

Digital images are conventionally coded in the red–green–blue (RGB) color system. If the chromatic scale is somehow connected to the *H* value, as in the present work, it is convenient to convert the images from RGB to HSV. The bottom part of Figure 3 reports the details of the color range input of the present work, which are used to verify the link between *L* and *H* and make the calibration in order to have a linear correlation as explained in the following.

**Figure 5:**
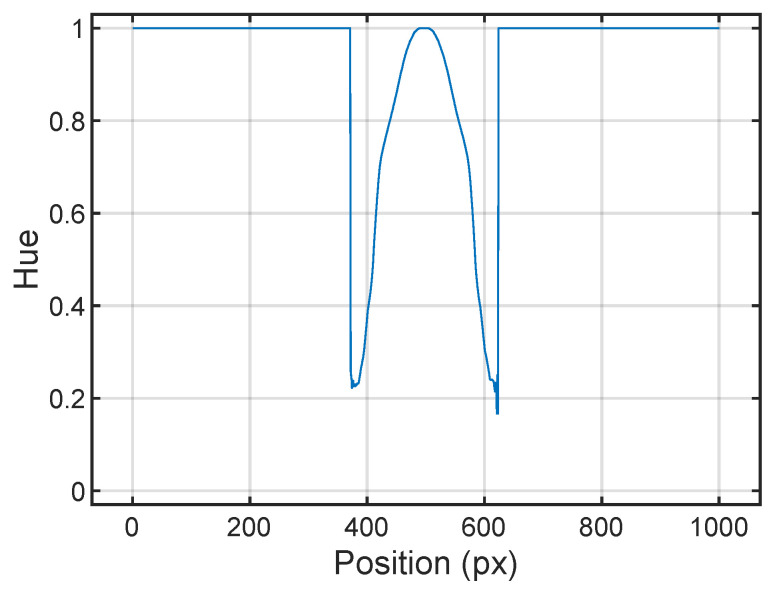
Discontinuity in *H*. Trend of the *H* value along the *x* direction in the correspondence of the center of sound map.

In this way, as can be seen in Figure 1, where the sound map is present, i.e., the *S* is nonzero (0<S≤1), the *H* channel would somehow represent the sound level calculated by the beamforming algorithm. The remaining part of the image is characterized by a zero value of *S* but by the same *H* as the red color, i.e., H=0°. A discontinuity in the *H* value at the border of the sound map occurs, as reported in Figure 5. In the present case, the −6 dB color is purple, corresponding to H=295° and equal to the discontinuity at the border.

The discontinuity in *H* visible in Figure 5 is due to the transition from a colored area (the sound map) to a black and white area. In the latter, *H* is not well defined, and an arbitrary value of H=1 is assigned to it in the RGB to HSV conversion process. The saturation filter described in Section 3.2 was implemented in order to handle this discontinuity. The strategy used to correctly extrapolate the sound level (*L*) from the sound map is to evaluate the transfer function *F* between *H* and *L*, as defined in Equation (1). In the discussion section, a different approach is tested and compared to the chosen one.

(1) H=F(L)

The result of the evaluation of F(L) is shown in Figure 5. The reported *H* is scaled to the interval between 0 and 1 in order to have a more intuitive representation. Then, it is inverted to let the maximum of *L* correspond to the maximum of *H*.

**Figure 6:**
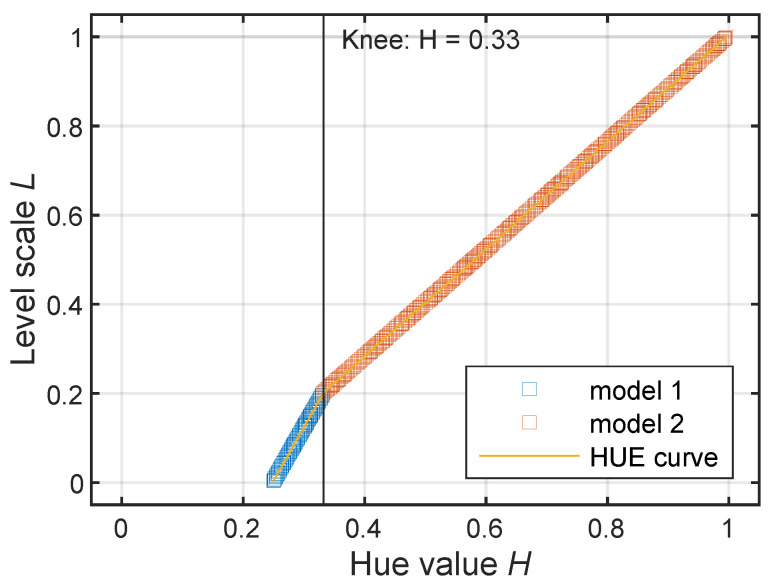
G(H), inverse transfer function between *H* and *L*, in yellow; in blue and orange, the two models $g_{1}\left(H\right)$ and $g_{2}\left(H\right)$.

Figure 5 highlights how F(L) is not linear but has a knee around i = 0.2. An inverse relation is then needed to determine the level from the hue value *H*. In fact, performing a linear regression between the two quantities and inverting the relation would not be sufficient to retrieve the relation. The proper inverse transfer function G(H) is more conveniently derived directly from the data. The trend of *L* based on the value of *H* derived from the image in Figure 3 is reported in yellow in Figure 6. In blue and in orange are also reported the two linear models calculated at each side of the knee ($g_{1}\left(H\right)$ and $g_{2}\left(H\right)$). The G(H) is then a piecewise-defined function according to Equation (2).

(2) $$G\left(H\right)=\left\{\begin{array}{c} g_{1}\left(H\right)\;for\;H<H_{knee}\\ g_{2}\left(H\right)\;for\;H\geq H_{knee} \end{array}\right.$$

where $H_{knee}$ is the *H* value corresponding with the knee and $g_{1}\left(H\right)$ and $g_{2}\left(H\right)$ are the two models estimated before and after the knee. The obtained G(H) is general and has validity for all the images produced with the same AC software and the same color scale regardless of the dynamic range selected. Thus, it has been used to convert the *H* value from maps to the level value for all the analyzed images.

As the *H* interval covered by the provided color scale in Figure 3 (0.25–0.99) is smaller than the typical *H* interval found in the output images like the one in Figure 1 (0.23–1.00), the obtained calibration curve G(H) refers to a limited level scale that, if applied to the data extracted from the original image, would produce level values bigger than 0 and lower than −6 dB. This first attempt to correlate *H* and *L* needs a further step where the G(H) is scaled for a correct reproduction of the required level interval.

### 3.2. Conversion to HSV and Initial Filtering

**Figure 7:**
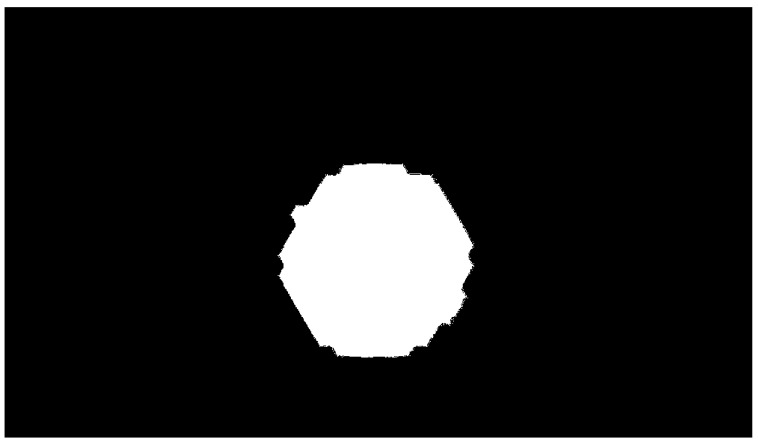
Saturation mask in which the zero S pixels are represented in black.

Some preliminary operations for the image are needed before executing the extraction process. The original image from Figure 1 is firstly cropped to carve out the user interface which can interfere with the process. The resulting cropped image is a matrix *y* by *x*, where *x* is the width and *y* is the height, with dimensions of 1000 × 575 px. Then, a first filtering stage consists of implementing a saturation mask that selects the pixels with a nonzero value of S, as reported in Figure 7. This stage allows excluding the black-and white part of the image and avoiding the discontinuity in *H* reported in Figure 5. After this step, *H* is transposed to the 0–1 interval. The *S* channel filter is then used to set the black-and-white area of the image to *“Not a Number”* value to exclude them from the fit.

### 3.3. Artifacts Removal

**Figure 8:**
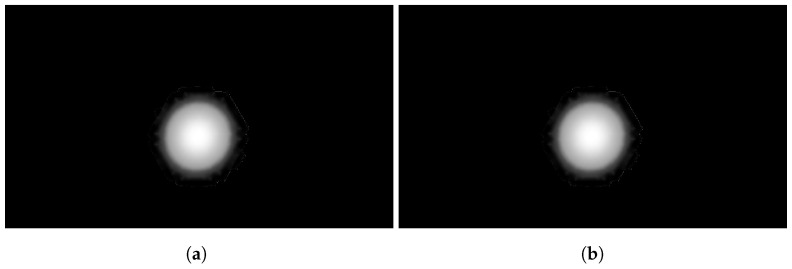
Blurring effect. (**a**) A raw image with spurious peak values. (**b**). Result of artifact removal with the blurring function B=5.

Due to the manipulation described in the previous subsection, spurious pixels can emerge at the border of the sound map, as shown in Figure 8a. Those are likely due to the original compression of the image that generates *H* oscillations at the border of the colored area. A blurring function (*B*) is applied in order to clean up those artifacts and obtain a clear image as in Figure 8b, according to the implemented code that follows:

%% *Artifacts removal*
B = 1;
h = ones(2 ∗ B + 1, 2 ∗ B + 1); % *filter matrix*
weight = **sum**(**sum**(h)); % *coefficient weight*
h = h/weight; % *weightening of coefficient filter*
**for** i = 1: **length**(dati)
      dati(i). Clean_image = imfilter(dati(i).Raw_image,h);
**end**

in which

- dati(i).Raw\_image is the original image with artifacts;
- dati(i).Clean\_image is the clean image after the blurring function.

**Figure 9:**
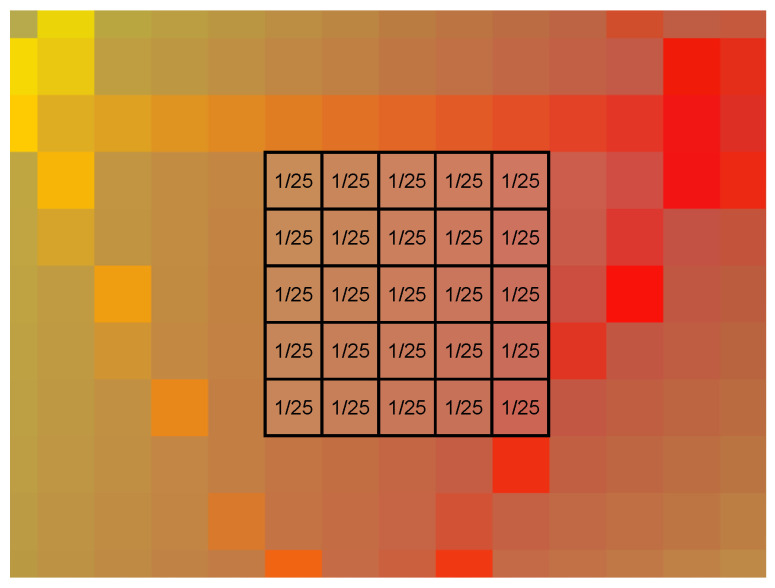
Blurring matrix with *B* equal to 2. A weight equal to one over the total number of elements is given to each element.

The blurring in the pixel $p_{ij}$ is applied based on the value of the near pixels inside the square of 2B+1’s long edge with a total number of elements n equal to (2B+1)2. The blurring matrix with B=2 is reported in Figure 9 where all the pixels are associated with a weight equal to 1/n. How the various intensities of the blur function affect the extracted data is studied in Section 4.

**Figure 10:**
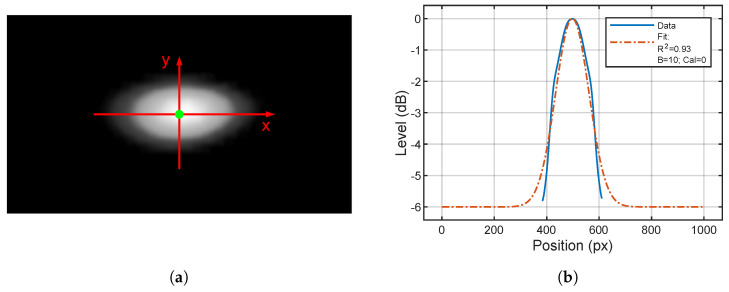
The fit process. (**a**) The identification of the fit axes: in green the maximum of *L*, in red the axes along the fits are performed. (**b**) The fit results along the *x* direction. $R^{2}$ is the coefficient of determination of the performed fit. Cal=0 stands for the calibration performed correlating the *H* interval to the actual dynamic range.

Once clear images are obtained, a fitting operation should be performed to extract parameters from the sound map. The first step consists of the identification of the maximum of *L*, indicated in green in Figure 10. Then, two Gaussian fits are made along the *x* and *y* direction on the axes passing through the maximum.

The fitting functions are non-normalized Gaussian functions as reported in Equations (3) and (4), respectively, for *x* and *y* axes.

(3) $$f\left(x\right)=C_{x}\exp\left(-\frac{{\left(x-x_{0}\right)}^{2}}{2c_{x}^{2}}\right)$$

(4) $$f\left(y\right)=C_{y}\exp\left(-\frac{{\left(y-y_{0}\right)}^{2}}{2c_{y}^{2}}\right)$$

where

- *x* and *y* are the positions along the *x* and *y* axes;
- $x_{0}$ and $y_{0}$ are the center of symmetry of the functions;
- $c_{x}$ and $c_{y}$ are the standard deviation dx (dy) of the function;
- $C_{x}$ and $C_{y}$ are the height of the functions.

### 3.4. Features Definition and Representation

Once the fits are performed, the modulus of distances *r*, in px, between the calculated center with coordinates ($x_{0}$, $y_{0}$) and the expected center with coordinates ($x_{S}$, $y_{S}$) of the sound source can be calculated with the simple relation reported in Equation (5).

(5) $$r=\sqrt{{\left(x_{s}-x_{0}\right)}^{2}+{\left(y_{s}-y_{0}\right)}^{2}}$$

The coordinates of the sources are obtained manually by the operator using the original AC image. Then, the total standard deviation dr is calculated from the original dispersion of the two functions following Equation (6).

(6) $$\sigma_{r}=\sqrt{\sigma_{x}^{2}+\sigma_{y}^{2}}$$

**Figure 11:**
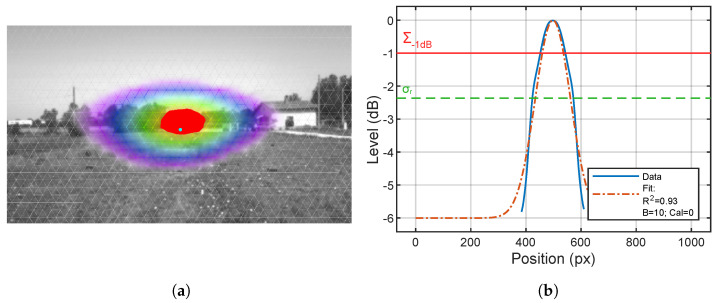
Source overlapping. (**a**) Example of positive overlapping of real source position, in cyan, and $\Sigma_{-1dB}$ in red. (**b**) Comparison between the section at −1 dB and the section corresponding to σ which is equal to the $\sigma_{r}$ value. $R^{2}$ is the coefficient of determination of the performed fit. Cal=0 stands for calibration performed correlating the *H* interval to the actual dynamic range.

Finally, $A_{-1dB}$ is calculated as the area corresponding to H>−1dB. $OVL_{-1dB}$ and $SC_{-1dB}$ are obtained by comparing the $A_{-1dB}$ mask with the position of the real source: $OVL_{-1dB}$ multiplies the masks and then counts the remaining pixels, while $SC_{-1dB}$ carries out the ratio between the dimensions of the two masks. An example is reported in Figure 11a, with the area corresponding to $A_{-1dB}$ in plain red and the surface of the real source in cyan. Figure 11b shows the corresponding curve of *H* along the x direction and two horizontal thresholds corresponding to the surface $A_{-1dB}$, in a continuous red line, and to $\sigma_{r}$, in a green dashed line. $\sigma_{r}$ corresponds to a level of −2.4 dB, meaning that the area corresponding to the parameter $\sigma_{r}$ is greater than $A_{-1dB}$.

**Figure 12:**
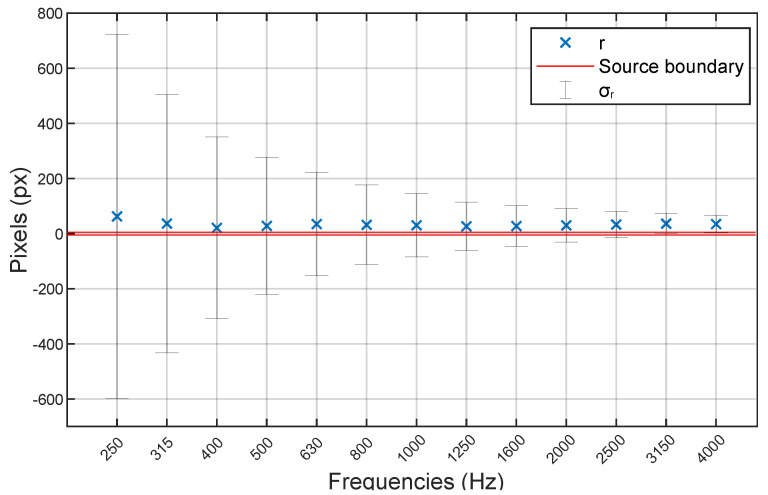
Representation of the results in the third-octave band for a single measurement with a source distance of 45 m. Red line is the physical dimension of the source, blue crosses are the *r* in each band, and $\sigma_{r}$ are the error bars.

As AC measurements may produce different results depending on the different frequency band, sound maps should be produced for at least the third-octave bands between 250 and 4000 Hz. A brief representation of the result for a single measurement that would include all these bands is shown in Figure 12, where for each frequency band, the distance from source *r* and the uncertainties, in terms of standard deviation $\sigma_{r}$, are reported. Distance can be eventually converted into meters by knowing the conversion factor from pixel to meter, which is unique for each distance.

$A_{-1dB}$, $OVL_{-1dB}$ and $SC_{-1dB}$ can be calculated for each frequency, distance source AC, as the example reported in Table 1 for distance 45 m. When $A_{-1dB}$ is very large, overlapping is very likely and therefore $OVL_{-1dB}$ becomes a simple true or false check. For the example, as shown in Table 1, up to 2 kHz, the ratio between the $SC_{-1dB}$ areas is very small, while for higher frequencies, $A_{-1dB}$ is very small; thus, overlapping becomes more difficult.

## 4. Discussion

In this section, we investigate and discuss how the tunable parameters affect the results of the procedure. These are the intensity of blurring (*B*), introduced in Section 3.3 for artifact removal, and the calibration method of the H−i relationship (Section 3.1).

A variation of *B* could affect the fit function, as the more intense the blurring is, the more artifacts are removed from the image, with a consequent lowering of the fault rate. The fault rate is defined as the percentage count of the fit functions that failed during the overall process. It has been noted that this can happen if an image is too influenced by the presence of artifacts, which is detected by the $R^{2}$ value of the fit being greater than 1 or less than 0. In these cases, a bigger *B* would save the data and allow its processing with the procedure.

The downside of the blurring function lies in the fact that modifying the image also involves changing the dispersion ($\sigma_{r}$). It is then important to analyze how *B* affects the results to evaluate its pros and cons and report the best choice obtained.

**Figure 13:**
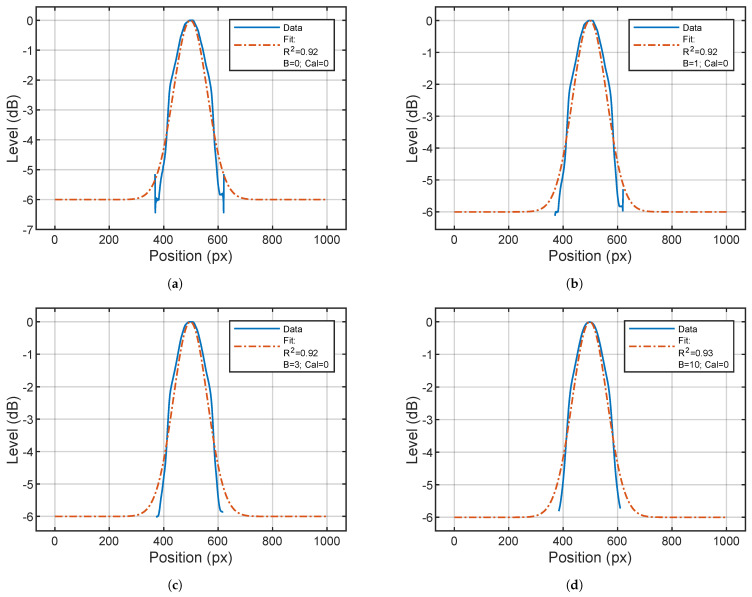
Example of fits over the same image on the *x*-axis while changing *B*. The source is at the center of the image. Cal=0 stands for calibration performed correlating the *H* interval to the actual dynamic range. (**a**–**d**) are produced with an increasing value of *B*, respectively 0, 1, 3, and 10. As can be seen, the artifacts diminish while *B* increases.

The effects brought by the variation of *B* are investigated by performing the computations with different intensities of *B* = 1, 2, 3, 5, 10 and then comparing the outcomes with the original image (B=0). The fitting results are reported in Figure 13.

In Figure 13, the oscillations occurring just below 400 px and just above 600 px are indicative of the artifacts present in the original images. The increase in *B* clearly reduces the intensity of the artifacts without visually altering the general trend of the figure and the $R^{2}$ of the fit.

Another significant effect of the variation of *B* can be visible in the fault rate, as reported in Table 2. The fault rate is evaluated through the value of $R^{2}$ on the different singular axis and, as faults can happen on both of them, the total fault rate is the union of both. A measurement is considered as “faulted” if one of the following conditions is encountered: $R_{x}^{2}>1$, $R_{y}^{2}>1$, $R_{x}^{2}<0$ or $R_{y}^{2}<0$. Each of these conditions corresponds to clear malfunction symptoms of the fitting process. The analysis is performed over all 297 images, and the percentages represent the number of images that failed the fit.

The calibration between *H* and *L* set in Section 3.1, and based on the chromatic scale (Cal=1) provided by the manufacturer’s software, can also have an influence on the results. Its effect is here tested and compared to an alternative approach (Cal=0), which is a simpler method consisting of correlating the *H* interval to the actual dynamic range (i.e., −6–0 dB) and assuming a priori that the relationship between *H* and *L* is linear.

**Figure 14:**
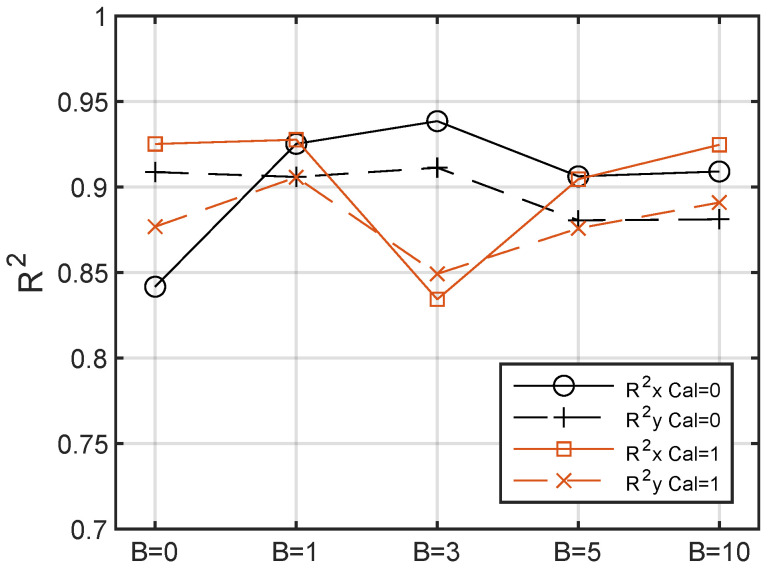
Averages of the $R_{x}^{2}$ and $R_{y}^{2}$ over all images as a function of B. “Cal=0” stands for calibration assuming linearity of the color scale and “Cal=1” stands for the calibration based on the chromatic scale.

Table 2 shows that when *B* increases, the fault rate decreases from 7% for the raw images to 1.4% with the highest *B* value. Calibration, on the other hand, does not significantly affect the fault rate, resulting in very similar results between Cal=0 and Cal=1. Figure 14 also shows that the moderate variation in the $R_{x}^{2}$ and $R_{y}^{2}$ values is caused by the introduction of blurring, confirming that it produces little to no modification to the relevant part of the signal.

**Figure 15:**
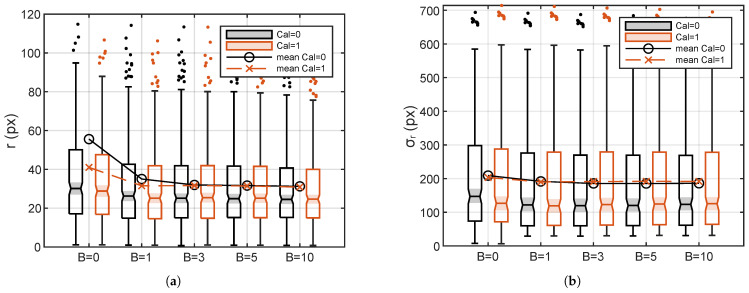
Box plot of the combined effects of *B* and calibration on *r* (**a**) and $\sigma_{r}$ (**b**).

Figure 15 reports *r* and $\sigma_{r}$ averaged over all the images as a function of *B* for the two calibration strategies. Calculations are based on all the images with successful fit in the two directions (*x* and *y*). It comes out that the change in *B* also affects the average *r* and its dispersion $\sigma_{r}$. Both the distance *r* between the real sources and the extrapolated position and the size of the sound map ($\sigma_{r}$) decrease while increasing *B*, confirming the advantage of the introduction of the blurring. The bigger variation in *r* is spotted at B=0, and negligible differences can be seen at B>1 for the two calibration strategies. Little variations are present between the two calibration results for $\sigma_{r}$, where Cal=1 leads to a slightly lower $\sigma_{r}$ for B=0 and causes a light increase for B>1.

*B* is confirmed to not significantly affect the results, while it could increase the accuracy. An increase of *B* also generates a little variation of $\sigma_{r}$.

Figure 14 and Figure 15 serve as examples, and the data reported in this study were extracted from sound maps calculated across various frequency bands. These maps were considered independent measurements intended to enhance data analysis. It is important to note that outliers and variations in *r*, $\sigma_{r}$ and $R^{2}$ are due to the inclusion of all obtained maps. Indeed, the map’s size is highly correlated with the wavelength of the corresponding frequency band. Other variations may arise from the different positions of the source in relation to the AC or from reflections induced by the height from the ground. A further exploration of these aspects can be conducted in future studies utilizing the developed tool.

## 5. Conclusions

The present work developed a methodology for extracting features from images with superimposed sound maps and provided parameters that will serve as the basis for future studies comparing sound maps calculated with different algorithms or acquired with different ACs.

The images used as input/tests in the present work have their sound maps computed using only the Delay and Sum (DAS) algorithm applied to AC acquisition in the free field. During the measurements, the AC was in a fixed position, while the known sound source was moved in height, distance and position.

The features extraction algorithm presented in this work starts from correlating the sound level scale (dB) to the color one (HSV). As preliminary steps, the image is cropped and transformed from RGB to HSV, and then a saturation mask is applied in order to exclude the non-relevant parts. While extracting the HUE data from the images, these steps simultaneously highlight the original defects due to the compression and quantization of the image. Artifacts also emerge, and they are removed with a blurring function (*B*). Finally, the real features extraction consists of the identification of the maximum point in the sound map and two subsequent Gaussian fits along the *x* and *y* directions passing through the maximum. The final defined data are the modulus of distances (*r*) between the calculated center and the expected center (in px) and the total standard deviation ($\sigma_{r}$), which was calculated from the original dispersion of the two functions. The two parameters numerically provide the position of the point of emission in the image and allow evaluation of the precision of localization. They are easily comparable with those calculated from different sound maps.

The effects on the algorithm’s output brought by the tunable parameters’ intensity of blurring (*B*) and calibration of HUE level relationship have been investigated and discussed. More artifacts are removed from the image with bigger *B* with a consequent lowering of the fault rate; i.e., the percentage count of the fit functions that failed on the overall process. The cost of the blurring function is a very marginal loss in the overall number of pixels where the fit is applied. The best value of *B* should be the one that maximizes the cost–benefit ratios, i.e., the value that improves the results without altering the results too much. The analysis indicates that B=1 is the most appropriate because, compared to the case of B=0, it is the first value of *B* that reduces the error rate of the fit to close to 2%, minimizing the waste of data and having a higher reliability of the results. B=3 significantly improves r estimation while slightly reducing $\sigma_{r}$, and it yields excellent $R^{2}$ fit results.

The authors do not exclude that a different calibration for the *B* coefficient may be required with images taken with other instruments. The results obtained show that any value of *B* improves the output compared to the case of not using blurring (B=0), and that whatever *B* is chosen, this does not negatively affect the results of the algorithm. As a result, the effect of calibration was limited. As a refined calibration is desirable in case of color scales with more pronounced non-linearity, the authors suggest performing a preliminary evaluation before the first application.

The proposed features extraction algorithm is deliberately very simple to implement, easy to reproduce and has a fast computation. While these qualities are required for elaborating a big number of images in a series, it can have some critical issues if not used under specific conditions. At present, the algorithm works on images with a single predominant source, as it does one fit per image. The multi-source case will be the aim of future developments investigating the separation of two known sources with ad hoc masks over the search domain. Another aspect to be further investigated, still due to the simplicity of the code, is that fits are performed only along the axes with possible discrepancy occurring for sources generating high asymmetrical sound maps.

In order to compare sound maps elaborated with several algorithms and analytically investigate their source localization efficacy in upcoming works, more operator-oriented parameters have been defined: $A_{-1dB}$ is the surface of the map from the max level to 1 dB below it, $OVL_{-1dB}$ is the percentage of overlapping between the real source dimension on screen and the $A_{-1dB}$ mask, and $SC_{-1dB}$ is the surface comparison between the real source dimension and the $A_{-1dB}$ dimension in percentage. They further support the analysis of beamforming algorithms that do not create symmetric images like DAS commonly does. A good algorithm, with high accuracy and precision, is expected to have high $OVL_{-1dB}$ and $SC_{-1dB}$ values and low *r* and $\sigma_{r}$ values. DAS has been shown to generate a large sound map, and consequently large $A_{-1dB}$, which means a higher probability of gaining a positive overlap and a reduced surface comparison. The applicability of the method can be much broader, because it could be used for generic applications in other sectors, such as the infrared output maps of thermal imaging cameras. This is particularly the case for closed proprietary software that only takes out hit maps and extracts data from old software and/or machines that only provide images. In these eventualities, the procedure can be easily adapted by only adjusting the dB-HSV correlation.

## Figures and Tables

**Table 1:** Overlapping results.

Frequencies (Hz)	$\Sigma_{-1\mathrm{dB}}\;\left(\mathrm{px}\right)$	${\mathrm{OVL}}_{-1\mathrm{dB}}\;\left(\%\right)$	${\mathrm{SC}}_{-1\mathrm{dB}}\;\left(\%\right)$
250	244,619	100	0.03
400	134,034	100	0.05
500	71,262	100	0.10
315	47,042	100	0.15
630	28,732	100	0.24
800	16,608	100	0.42
1000	10,936	100	0.63
1250	6280	100	1.10
1600	4373	100	1.58
2000	2964	67	2.33
2500	1788	0	3.86
3150	1048	0	6.58
4000	763	0	9.4

**Table 2:** Fault rate and singular axis fault rates evaluated over $R^{2}$ at different *B* values for all the figures.

	B=0	B=1	B=3	B=5	B=10
	(%)	(%)	(%)	(%)	(%)
Cal=0− Fault rate on *x*	5.1	1.0	1.0	1.0	1.0
Cal=0− Fault rate on *y*	4.1	0.3	0.3	0.3	0.7
Cal=0− Fault rate	7.2	1.4	1.4	1.4	1.7
Cal=1− Fault rate on *x*	4.8	1.4	1.0	1.0	1.0
Cal=1− Fault rate on *y*	4.8	1.0	0.3	0.7	0.3
Cal=1− Fault rate	7.9	2.1	1.4	1.7	1.4

## Data Availability

The raw data supporting the conclusions of this article will be made available by the authors on request.

## Abbreviations

The following abbreviations are used in this manuscript:

AC	acoustic camera
UAV	Unmanned Aerial Vehicles
DAS	Delay and Sum
EVOB	eigenvalue-optimized beamforming
HSV	hue saturation value
OVL	surface overlapping
SC	surface comparison
RGB	red–green–blue
